# Labeling of Genetically Modified (GM) Foods in Peru: Current Dogma and Insights of the Regulatory and Legal Statutes

**DOI:** 10.1155/2022/3489785

**Published:** 2022-05-12

**Authors:** Jaime Delgado-Zegarra, Aldo Alvarez-Risco, Carmen Cárdenas, Massiel Donoso, Stephanie Moscoso, Brenda Rojas Román, Shyla Del-Aguila-Arcentales, Neal M. Davies, Jaime A. Yáñez

**Affiliations:** ^1^Instituto de Consumo, Facultad de Ciencias Administrativas y Recursos Humanos, Universidad de San Martin de Porres, Lima, Lima, Peru; ^2^Carrera de Negocios Internacionales, Facultad de Ciencias Empresariales y Económicas, Universidad de Lima, Lima, Peru; ^3^Gerencia Corporativa de Asuntos Científicos y Regulatorios, Teoma Global, Lima, Peru; ^4^Universidad Cristiana de Bolivia, Facultad de Medicina Humana, Santa Cruz de la Sierra, Santa Cruz, Bolivia; ^5^Escuela Nacional de Marina Mercante “Almirante Miguel Grau”, Callao, Peru; ^6^Faculty of Pharmacy & Pharmaceutical Sciences, University of Alberta, Alberta, Canada; ^7^Vicerrectorado de Investigación, Universidad Norbert Wiener, Lima, Peru

## Abstract

The COVID-19 pandemic has affected and afflicted human lives and been a transformative catalyst leading to closure of many companies, disrupting mental health, and reducing access to food and exacerbating food insecurity. This presents an opportunity to reflect on and examine genetically modified (GM) foods and their effective legislative regulation for the benefit of consumers. This review presents a detailed analysis of GM foods' regulation in Peru and the analysis of certain specific cases that show the need for greater regulation of the industry.

## 1. Introduction

The COVID-19 pandemic has altered society affecting business including tropical small-scale fishing communities [1], e-commerce and trade [2], freight transport [3], retail investors [4], and agricultural production [5]. It has also been reported to have significant effects on workers [6–11], regulations [12–14], entrepreneurship [15, 16], education [17–19], intellectual property [20], firms [21], prices [22, 23], tourism [10, 24, 25], and the general public [13, 14, 26–32]. As with previous pandemics, COVID-19 has led to various limitations on access to essential products being brought about [33]. COVID-19 generated a negative impact on access to food, which has been recognized in the scarcity or lack of availability of certain products in grocery stores. This food limitation raises some questions regarding the use of biotechnology to address food shortage problems with genetically modified (GM) foods. Despite the need and supply chain pressures, some regulations and process control are still pending in terms of environmental products [16, 17], which contributes to sustainable development [34–36]. This article presents an analysis of Peru's compliance with the current regulations related to GM foods' labeling. As the need for the labeling of GM foods has been raised, we must make decisions based on a critical analysis of sometimes disparate opinions. However, one of the decisive factors to aid in the discussion between the obligation, or not, to correctly label foods that have been exposed to genetically modified organisms (GMOs) in their production is to understand consumers thoughts and concerns. Consumers usually know very little about the quality of food and the process of making it. For this reason, it is essential to follow the United Nations guidelines that recommend that every effort be made to maintain sustainable development [37]. It is necessary that all the people of the planet have access to a satisfactory level of social, economic, human, and cultural development so that resources are used reasonably with a goal of preserving species [38].

Some food consumption trends have paradoxes between the production and growing consumption of transgenic foods or GMOs [39], on the one hand, to respond to world overpopulation problems and, on the other hand, there is the constant concern of environmental impact. The ecological behavior is used to define what is proenvironmental and is usually related to behaviors in favor of the environment; therefore, consumer ecological factors to one whose purchasing behavior is influenced by a concern for the environment must be addressed [40]. Similarly, companies responsible for the environment develop “green” brands, whose processes are aimed at clean production, through environmentally friendly marketing, and that a consumer will trust that the product is considered healthy [30, 41–52]. However, to know if a food is friendly to the environment, the consumer needs to know whether that food contains transgenic ingredients to be able to decide whether to consume it or not depending on preferences as an ecological or proenvironmental consumer.

The Food and Agriculture Organization of the United Nations estimates that almost 690 million people went hungry in 2019—up by 10 million from 2018 and by nearly 60 million in five years [53]. The staggering aforementioned statistics are what served as sustenance for the further development and increased presence of transgenic ingredients and foods, including intentionally manipulated plant, animal, or other entities [54]. Global acceptance of GMOs varies due to several factors; first, it depends on where in the production chain a survey is taken. For example, farmers who grow GM foods are customers of the companies that sell GM seeds and have made decisions to accept GM foods [55]. There are many transgenic seeds available at low cost, and the certified nongenetically modified products represent a market niche for consumers [55]. Second, the opinion and acceptance of transgenic foods vary between countries due to the country's regulations. Open and public discourse and debate about the effects on health, the environment, and the consumer's rights to information can vary widely.

Unfortunately, the lack of scientific understanding and awareness of transgenic foods has led to low consumer confidence since traditionally, the perception of a person on a specific topic is based not only on information but also on trust, beliefs, perceptions of risks and benefits, and personal development of how external information is processed and evaluated. For example, in Europe, transgenic crops have often become stigmatized as the basis for explaining everything terrible about modern agriculture and food, large corporations' excessive growth, inequitable economic development, globalization, and growing inequality [55], creating an adversarial debate on GM foods in Europe an issue that is not based on scientific facts, but more on a political agenda. Whatever the reason, in Europe, one does not find transgenic food on the market since it is highly regulated, but there is a discrepancy since, for example, most of the soy that is regularly imported into the European Union is genetically modified and ends up being used as animal feed in Europe. However, most consumers choose to ignore this dichotomy [56, 57].

The other side of the coin is the United States, where the use of GM foods without the need for labeling is highly politicized, and voluntary GM-free labeling schemes have become a niche market with high growth economic potential [58, 59]. Interestingly, China has taken a slightly more central position in seeking to promote the need to use modern technologies (including transgenic crops) to foster more productive and sustainable agriculture in a society that actively participates in the discussion on its pros and cons, but overall listening to the concerns and preferences of consumers. China has developed a policy framework on this issue that focuses on supporting the right of consumers to make individual decisions, even if they are not based solely on “scientific facts” and considers broader thoughts on ways of life and what innovations are considered desirable or problematic by society [59].

The inclusion of these democratic elements and freedom of choice in the discourse should assist the veracity of arguments and contribute to sharing the responsibility for future developments among all society elements, including science as an integral part of it [60]. The position China has taken has led to various studies on Chinese consumer perception [61]. It has been determined in China that 57% of the Chinese public do not know that they usually consume or purchase GM products or products containing GM ingredients. It is noteworthy that 78% of the Chinese public find acceptable foods labeled as non-GM, indicating that most would prefer to know and consume foods without GM ingredients [61]. However, when explicitly asked if labeling were to be mandatory, how would their preferences change? In that case, 57% would accept foods without GM labeling, indicating that they do not really care if they contain GM ingredients, while 41% would accept products such as meat and oil condiment that contain GM ingredients and are labeled as such [61].

In Hispanic countries, the discussion has evolved out of an environmental centric position that serves as a preamble to discuss transgenic foods. It has been observed in Argentina and Spain that some investigations demonstrate the intention of consumers to buy environmentally friendly products [62, 63] apprehension about the label, guarantee, and origin of the product; the degradation of natural resources; the dominance of productive systems that privilege the profit motive over socio-environmental concerns; and the loss of sense of collective well-being [64]. The price variable and ignorance of scientific and health concerns continue to be, among citizens in the middle and lower economic strata , an interference factor in their purchase [65]. This has led to creating an ecological label so that the consumer is clearly made aware if a product has a reduced environmental impact throughout its life cycle and provides consumers with accurate, nonmisleading, and scientifically based information on its environmental impact [66].

The relevancy of creating an eco-label has been empowered as consumers demand environmentally friendly products from the market that often incorporate the words “ecological,” “green,” “natural,” or “recycled” into their products [67]. In this line, the International Organization for Standardization (ISO) creates an eco-labeling standard ISO 14024: 2018 [68] to generate confidence in the end consumer who seeks to know about what food or product is composed [69] or how it has been manufactured [70] and prefers products that on their labeling have environmental declarations such as natural, recyclable, ecological, and low-energy recycling, among other terms [71]. GM foods have the corresponding sanitary records in the countries in which they are consumed, which shows that health authorities support such foods' safety; likewise, countries have requirements for companies to declare GM ingredients on their labels. For these reasons, we pose several questions that consumers may also ask for further clarification: Why are consumers afraid of evaluating GM labeling when they have the sufficient and credible information? Is it that the safety issues and concerns still remain? Are the health records delivered without carrying out a corresponding comprehensive evaluation of these products' safety and benefits? Does this happen in all countries, or does it vary between different countries where the same product may be sold? Should each country legislatively require companies to declare everything on their labels, or should they be limited to a minimally acceptable amount of information to the consumer?

These provocative questions seek to show that there is no harmony between the expectation and right of consumers to have this information provided and the attitude of some companies not to willingly disclose it. This could be affecting the trust in consumers and their loyalty towards the brands of products that contain GMOs and do not declare it. There are some excellent reviews, innovative position papers, and various contemporary studies reported in the literature, which provide an international context of perceptions of consumer preference of genetically modified foods using various approaches. These studies include the importance of social media, various belief models, quality to risk perception, and the use of enhanced labeling information [72–82].

The current article is focused on an analysis undertaken based on a comprehensive literature review of the legal aspects of Peruvian legislation related to GMOs and GM labeling. The aim of the current study was to present the context of the Peruvian legal doctrine with respect to GMOs and provide a comprehensive analysis of the regulatory environment and possible changes required. In this article, we carry out a legal analysis based on GM food labeling's legal advance in Peru. The legal documents that have established the declaration of components in GM foods are presented, and the components of the Consumer Protection and Defense Code are described, as well as various current examples of products that openly declare GM components on their label when sold in a country other than Peru, but when sold in Peru, that information has not been openly provided. Finally, some legal cases are presented that also exemplify the need for regional and ideally global standardization of GM components' declaration in the food products that are retailed. In Peru, this information should be made easily accessible to the consumer, similarly to how it is undertaken in the United States via a QR code as its current regulation provides.

## 2. Legal Framework

Genetically modified or transgenic foods have evoked and generated various concerns among international organizations, consumers, and the scientific and academic world [83–86]. Initial concerns were raised because of possible health implications [87–93], mainly due to the lack of long-term chronic consumption studies [89], and possible detrimental environmental effects [94]. However, it has been observed that transgenic crops have also caused resistance to some herbicides and insecticides, which may further cause possible risks to health and ecosystem diversity, resulting in ecosystem disruption via altering organisms and causing species resistance [95]. Furthermore, damage and alteration of soil microbes, reduction in pollinator populations, and natural processes that lead to a reorganization of food chains have also been observed [96].

In addition, a lack of detailed information may deprive consumers of an informed decision at the time of purchase. Informed consent, is protected in Peru under the Consumer Protection and Defense Code (CPDC) [97] as an axis principal. This protection is expressed in the very purpose of this important legal instrument, under the following terms: “This Code has the purpose that consumers have access to suitable products and services and that they enjoy the rights and effective mechanisms for their protection, reducing informational asymmetry, correcting, preventing, or eliminating behaviors and practices that affect their legitimate interests. In the social market economy regime established by the Constitution, protection is interpreted in the most favorable sense to the consumer, following the provisions of this Code” [97].

However, some companies are reluctant to declare GMOs' presence in their products, despite having an express provision that orders it. Article 37 of the CPDC provides that “Foods that incorporate genetically modified components must indicate them on their labels” [97]. There is no reason why the food industry does not inform consumers about the content of transgenic elements or inputs that they have been using in the food they sell in the Peruvian market, as this constitutes a violation, even in the State's Political Constitution itself [98] that establishes in its Article 65 that “The State defends the interest of consumers and users. For this purpose, it guarantees the right to information about the goods and services available for consumers in the market. It also ensures, in particular, the health and safety of the population” [98].

Also, the CPDC has established a set of fundamental principles concerning the right of information available to consumers.

1. Principle of transparency: in acting in the market, suppliers generate full accessibility to consumers' information about the products or services they offer. The information provided must be truthful and appropriate following this Code
2. Asymmetry correction principle: consumer protection regulations seek to correct distortions or bad practices generated by informational asymmetry or the situation of imbalance that occurs between suppliers and consumers, whether in contracting or in any other relevant situation, that place the latter at a disadvantage compared to the former when acting in the market
3. Proconsumer principle: in any field of its action, the State exercises a protective action in favor of consumers. In case of insurmountable doubt in the sense of the norms or doubts in the contracts' scope by adhesion and those concluded based on general contracting clauses, it must be interpreted in a more precise sense to the consumer

### 2.1. The Current Consumer Landscaper in Peru

- Interpretation of the consumer protection and defense code

Parts of the food industry have employed an argument that the Code is confusing and that there were inaccuracies in the established deadlines, and thus, they intend to claim that the labeling of GMOs has been suspended or rejected. For example, the third final complementary provision of the CPDC states that “within a period of one hundred and eighty (180) calendar days from the entry into force of this Law, the executive branch issues the regulatory provisions of what is provided in Article 37” [97]. The absence of specific regulations regarding GM foods does not mean that consumers do not have the right to be informed about the products' content in the market by suppliers; in other words, if a product contains GMOs, the label must report it. This is in line with the National Institute for the Defense of Competition and the Protection of Intellectual Property (INDECOPI) [99] which resolved, even before the CPDC was promulgated that “The transgenic condition of the inputs used in the elaboration of processed foods, constitutes relevant information to adopt an informed consumption decision within the framework of articles 5° literal b) and 15° of Legislative Decree 716 (102). The relevance of transgenic foods to these articles is based on the precautionary principle, due to which it is the consumers who must decide whether to assume the possible risks of their consumption. Consequently, suppliers are obliged to provide the requisite information to the consumer regardless of whether this information is part of the technical regulation of food labeling”.

In the fourth provision of the CPDC, the deadline after which all suppliers must disclose their products' GMO content indicates that “This Code enters into force thirty (30) calendar days from the day following its publication in the Official Gazette El Peruano, except for those indicated in the following paragraphs. Articles 36 and 37 enter into force one hundred and eighty (180) calendar days from the entry into force of this Code” [97]. This means that GMO labeling is mandatory from March 30, 2011, and has already been established in various resolutions by INDECOPI [100–102]. Consequently, all products containing transgenic elements or inputs should inform consumers of this on their product labels. However, more than nine years have passed, and many companies are still not providing this information in Peru.

(b) Examples of inappropriate labeling

**Figure 1:**
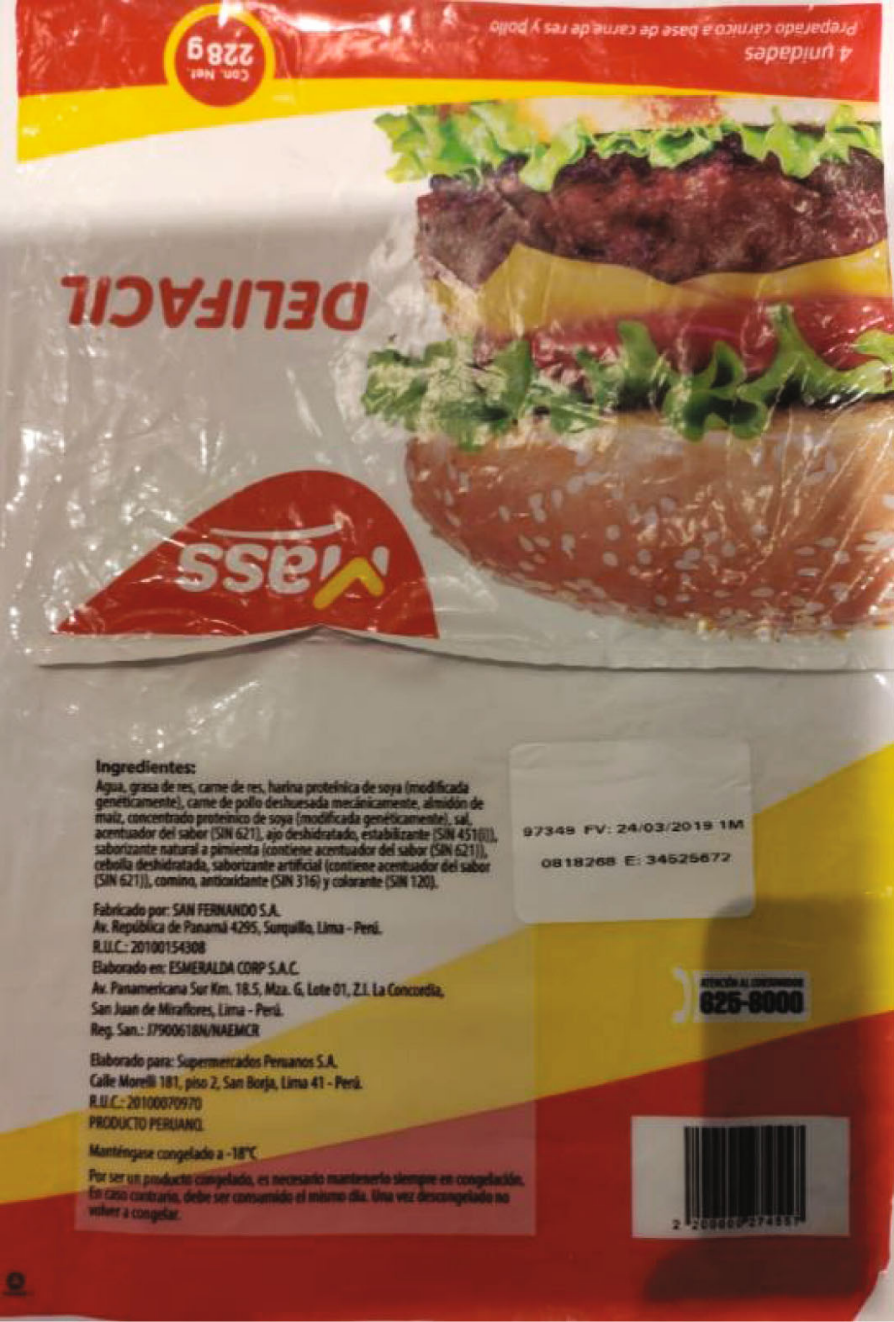
Delifacil brand hamburger label. The photo was taken for this study.

**Figure 2:**
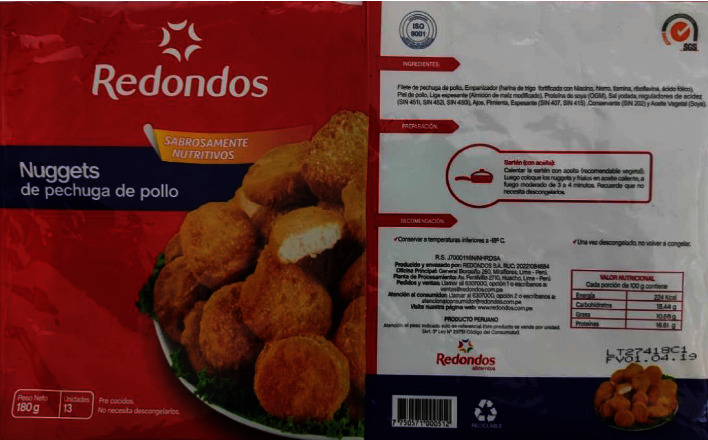
Label of Redondos brand chicken breast nuggets. The photo was taken for this study.

**Figure 3:**
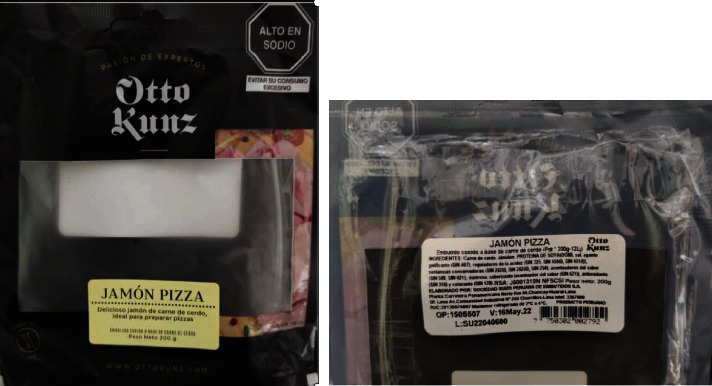
Otto Kunz brand pizza ham label. The photo was taken for this study.

Articles 1 and 2 of the CPDC indicate that the labeling must contain truthful, sufficient, easily understood, appropriate, timely, and accessible information [97]. However, companies use different and confusing names to declare the components “transgenic” to go completely unnoticed. Some companies declare the transgenic component with colors or contrast and in locations which may be difficult to perceive. Some examples are as follows:

- Delifacil hamburger (Figure 1) states the declaration of “transgenic” content but only do so in the list of ingredients and with the name of soy (genetically modified)
- The Redondos product (chicken breast nuggets) (Figure 2) shows very little in the ingredient ratio: soy protein (GMO); however, the acronym GMO is not defined or explained, and the consumer may not understand what it means. Unfortunately, in Peru, the size of the letters or information that must appear on the label is not defined in regulations, except for the warnings of law 30021 [103]. However, if the characters used are of dimensions that they are challenging to read without magnification by a consumer, this may violated the CPDC's right to provide this information
- The Otto Kunz product (pizza ham) (Figure 3) details in its list of ingredients and with a sticker the indication that it contains “GMO starch.” The term GMO, as such is an acronym which may not be easily interpreted by some consumers

These are representative and not exhaustive examples of labels that with additional detail could improve reading and comprehension of the intended consumers. For this reason, it is reasonable, necessary, and perfectly legal that INDECOPI has arranged “corrective measures” to amend these types of potential labeling ambiguities and guarantee the right of consumers to correct information, which complies with the minimum requirement of “suitability” to be perceived and easily understood.

(c) Some processes resolved by INDECOPI

The Specialized Chamber for Consumer Protection of the Tribunal for the Defense of Competition and Intellectual Property of INDECOPI, which is the last administrative instance, has already ruled in various processes on the validity of Article 37 of the CPDC and consequently the obligation that manufacturers must declare the GMO content of the products. We point out some specific cases here:

- Product “Choco Donuts”—Resolution No. 2304-2019/SPC-INDECOPI, the same one that imposes a warning and has a corrective measure so that it complies with adequate reporting
- Product “Chips Ahoy”—Resolution No. 2051-2019/SPC-INDECOPI, the same one that imposes a warning and has a corrective measure so that it complies with adequate reporting
- Product “Cheetos”—Resolution No. 2522-2019/SPC-INDECOPI, the same one that imposes a warning and has a corrective measure so that it complies with reporting appropriately
- Product “Pudin Royal”—Resolution 2651-2019/SPC-INDECOPI, the same one that imposes a warning and has a corrective measure so that it complies with the appropriate information

The Tribunal issued these provisions for the Defense of Competition and Intellectual Property of INDECOPI because these companies do not report in Peru the content of transgenics in their products. At the same time, in other countries, the GMO reporting in the label is mandatory, and specific regulations are written to do so, such as in New Zealand [104], Australia [104], Brazil [105], and the European Union [106]. These processes, together with others resolved by INDECOPI against the product “Bunge Soybean Oil” [107] and M&M and Snickers products [108, 109], highlight the imperative need for similar labeling of transgenics in Peru and that the information on the content of transgenics in food should be standardized.

Faced with these INDECOPI resolutions, the brand “Choco Donuts has communicated to the authority” the complete blocking of the product's commercialization at the national level and that this company will not attend to sales orders for the 38 gr Choco Donuts product. A withdraw of product from the market rather than a declaration of the GMO content on its label may be preferential to some companies. For its part, Chips Ahoy reports that it has substituted “chocolate-flavored chips” with transgenic soy lecithin content for a product that does not contain transgenic ingredients. It was also preferred to change the formulation of the product rather than declare the transgenic content on the labels. The requirement of compliance with the labeling of GM foods could impact the sales of these products due to a possibly negative perception from consumers that some companies may prefer to change their formulation or withdraw the products from the market, rather than labeling them GM.

(d) Pending judicial process

Currently, a company has filed a contentious administrative action against the Resolution of the National Institute for the Defense of Competition and the Protection of Intellectual Property (INDECOPI) [110] that orders compliance with Article 37 of the CPDC on the labeling of transgenics, arguing that this article is not yet regulated, which was rejected by INDECOPI in several resolutions that have put an end to the administrative instance.

However, filing a contentious administrative action does not imply suspending the obligation imposed by INDECOPI to consign the content of transgenics on the food labels, as it has indicated, which could possibly lead to fines imposed on companies for noncompliance. It is important to emphasize that Article 37 of the Consumer Protection and Defense Code is clear [97], and in the INDECOPI Court resolutions, it is established that regardless of the number of transgenic inputs that these foods might contain, they are obligated by the regulations to declare their presence in the labels. In the absence of regulations, INDECOPI has already established criteria that will help guide the most convenient way of labeling for easy and quick reading by consumers of GM ingredients' content.

(e) The double standard of labeling by the same companies

**Figure 4:**
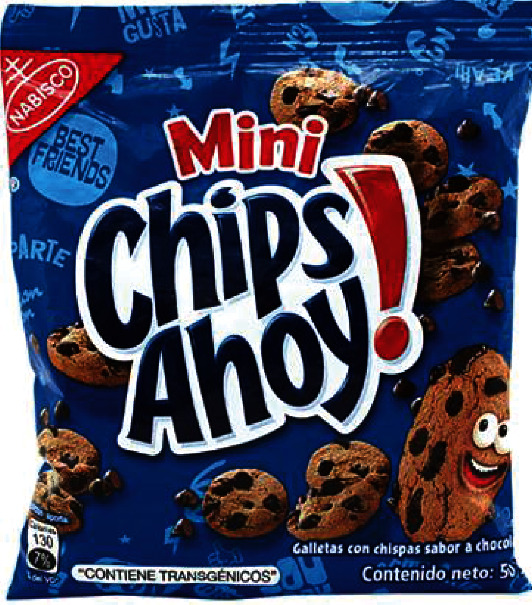
Chips Ahoy! Cookie label. The photo was taken for this study.

**Figure 5:**
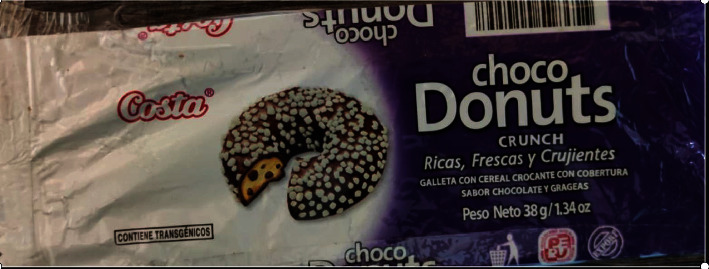
Choco Donuts brand cookie label. The photo was taken for this study.

A curious commercial phenomenon is that some of these companies installed in Peru, with production plants in Lima, manufacture and sell the same products with the same brands to neighboring countries such as Ecuador where they comply with the legend “CONTAINS TRANSGENICOS” on their labels. That is to say, for Peruvian consumers, these products do not provide information on their transgenic content, but neither do they detail whether it is a different GMO-free formula, which is possible but unlikely. A marketing strategy to have a competitive advantage is the case of the “Chips Ahoy” product manufactured in Peru, which complies with a declaration of the transgenic content clearly and prominently on the front of the label, but only when the product is exported to Ecuador (Figure 4), but when the product is for the Peruvian market, this information is not present in the label. This approach is mirrored by the product “Choco Donuts” manufactured in Peru, and when exported to Ecuador, it clearly and prominently states on the front of the packagethat the product contains transgenics (Figure 5), but when it is sold for the Peruvian market, that information is not displayed.

(f) Attempts to reduce GMO labeling requirements

There have been several attempts to modify the mandate of the CPDC [97], by establishing a limit of 2 or 3% so that only the percentage of transgenic is obliged to be declared on the product labels. Such modification is not legally feasible since, under the principle of hierarchy of norms, a regulation cannot modify or contradict a law and even less when this implies a reduction in consumer rights.

(g) No minimum thresholds are required for labeling

1. Any manufacturer must know each of the inputs, raw materials, or components of the product that it produces, and if it acquires them from third parties, it has the right and the obligation to require from them all the technical information on its origin, production methods, and components which is referred to as traceability
2. It is in the public domain that the main transgenic components that have been used in the industry are corn, soybeans, and their derivatives. Under these conditions, it would not be permissible for companies to buy from an unknown or clandestine supplier without requiring a technical sheet to ascertain the origin and characteristics of said product and whether it is or is not transgenic
3. In reality, it is possible that companies may know that the components they use to make their product are or are not genetically modified or transgenic. Demonstrable proof of this is that they either declare it on their labels or not when they sell their products in other markets than Peru
4. The limit of 2 or 3% that some sectors have been proposing is mutually exclusive to with the labeling because the law does not provide that it is only based on a certain percentage of transgenic components. The legal obligation is clear and straightforward; if a product contains transgenics, whatever its percentage, it must be consigned on its labels so that the consuming public is aware and makes their own informed decisions
5. Companies should know when their components are transgenic. Any refusal to declare such components is not due to technical aspects but to possible fear that their products will be rejected by some consumers who prefer not to consume this type of genetically modified food

## 3. Labeling Precisions

The labeling for transgenics is essential, and we detail some recommendations of the characteristics and details that they should incorporate in Peru, based on the resolutions issued by the last administrative instance of INDECOPI [111].

1. The size of the letter of the indication (TRANSGENIC) must be consigned on the main display face (front part) and be similar to that used by the supplier to report on the net content of the product, which is regulated in the “Norma Metrológica Peruana” (Peruvian Standard Metric) NMP 001: 2019 “Requirements for the labeling of prepackages” [112]
2. Its location must be such that it allows the consumer to identify this characteristic on the packaging
3. The color to be used must be noticeably different from the color used to label most packaging so that the message is not obscured within the label
4. The phrase to be consigned on the main display face must be one that allows consumers to comprehend the use of a transgenic input to produce their product. Therefore, the use of initials or any abbreviation that prevents or hinders the understanding of this characteristic is prohibited
5. For the precision of ingredients, the supplier must consign the word “TRANSGENIC” on the side of the component that has this characteristic

## 4. Conclusions

The labeling of transgenic foods in Peru is mandatory by the provision of Article 37 of the Consumer Protection and Defense Code and has been in force since March 30, 2011. Consequently, companies must comply with their obligation to declare it on their product labels, expecting that it does it in a transparent, visible, and prominent way on the front, as the INDECOPI Tribunal has established in various resolutions. The Peruvian government should through regulations enforce that the Consumer Protection and Defense Code be followed and that modifications to establish minimum limits of 2 or 3% transgenic content should not be made. Numerous cases in which INDECOPI has sanctioned companies that fail to label transgenic content have occurred, and penalties should be considered to ensure enforcement. There are many countries where labeling of transgenics is not even in discussion; we intend that our review can serve legislators in those countries as a framework to make adequate and englightened decisions in lieu of the consumers' protection and right to be informed.

## Data Availability

All the data relevant to the research can be found in the manuscript. Further information is available from the corresponding author upon the request.
